# Alarmins in Chronic Spontaneous Urticaria: Immunological Insights and Therapeutic Perspectives

**DOI:** 10.3390/biomedicines12122765

**Published:** 2024-12-04

**Authors:** Angela Rizzi, Federica Li Pomi, Riccardo Inchingolo, Marinella Viola, Francesco Borgia, Sebastiano Gangemi

**Affiliations:** 1 UOSD Allergologia e Immunologia Clinica, Dipartimento Scienze Mediche e Chirurgiche, Fondazione Policlinico Universitario A. Gemelli IRCCS, 00168 Rome, Italy; marinella.viola@policlinicogemelli.it; 2 Department of Precision Medicine in Medical, Surgical and Critical Care (Me.Pre.C.C.), University of Palermo, 90127 Palermo, Italy; federicalipomi@hotmail.it; 3 UOC Pneumologia, Dipartimento Neuroscienze, Organi di Senso e Torace; Fondazione Policlinico Universitario A. Gemelli IRCCS, 00168 Rome, Italy; riccardo.inchingolo@policlinicogemelli.it; 4 Department of Clinical and Experimental Medicine, Section of Dermatology, University of Messina, 98125 Messina, Italy; fborgia@unime.it; 5 Department of Clinical and Experimental Medicine, School and Operative Unit of Allergy and Clinical Immunology, University of Messina, 98125 Messina, Italy; gangemis@unime.it

**Keywords:** alarmins, IL-33, TSLP, IL-25, HSP, S100 proteins, EDN, defensins, chronic spontaneous urticaria, inflammation

## Abstract

**Background**: In the world, approximately 1% of the population suffers from chronic spontaneous urticaria (CSU), burdening patients’ quality of life and challenging clinicians in terms of treatment. Recent scientific evidence has unveiled the potential role of a family of molecules known as “alarmins” in the pathogenesis of CSU. **Methods**: Papers focusing on the potential pathogenetic role of alarmins in CSU with diagnostic (as biomarkers) and therapeutic implications, in English and published in PubMed, Scopus, Web of Science, as well as clinical studies registered in ClinicalTrials.gov and the EudraCT Public website, were reviewed. **Results**: The epithelial-derived alarmins thymic stromal lymphopoietin and IL-33 could be suitable diagnostic and prognostic biomarkers and possible therapeutic targets in CSU. The evidence on the role of non-epithelial-derived alarmins (heat shock proteins, S-100 proteins, eosinophil-derived neurotoxin, β-defensins, and acid uric to high-density lipoproteins ratio) is more heterogeneous and complex. **Conclusions**: More homogeneous studies on large cohorts, preferably supported by data from international registries, will be able to elucidate the intriguing and complex pathogenetic world of CSU.

## 1. Introduction

Chronic spontaneous urticaria (CSU) is a widespread and frequently debilitating skin disease that typically presents with recurrent episodes of pruritic urticaria and/or angioedema for a period of at least six weeks and at least twice a week [1]. CSU significantly impairs patients’ quality of life and poses a considerable challenge to clinicians due to its unpredictable course and resistance to conventional therapies [2,3]. Initially thought to be an eosinophil and basophil-related disease, it is now known that CSU is a mast cell (MC)-driven condition in which the activation and degranulation of cutaneous MCs lead to vascular permeability, vasodilatation, and inflammatory cell recruitment, which determine the clinical manifestation of the disease [4,5,6]. Concomitantly, a wide range of MC-associated pro-inflammatory cytokines has been identified as having pivotal implications in CSU pathogenesis, including interleukin (IL)-1β and IL-6, which drive the inflammatory process, and IL-31, commonly referred to as the “itch cytokine” [7,8].

Recent advances have shed light on the potential role of a family of molecules known as “alarmins” in the pathogenesis of several immunological-related conditions, including CSU [9]. Unlike pathogen-associated molecular patterns (PAMPs), which respond to external pathogens, alarmins are endogenous danger signals released by stressed, injured, or dying cells [10]. The alarmin family includes IL-33, IL-25, thymic stromal lymphopoietin (TSLP), heat shock proteins (HSPs), S100 proteins, eosinophil-derived neurotoxin (EDN), uric acid, and defensins [11].

These peptides and proteins act as early alarm signals which, after the interaction with specific receptors, activate the immune cells, subsequently triggering a cascade of inflammatory responses [9]. Specifically, alarmins induce neutrophil and macrophage release of inflammatory molecules including nitric oxide, tumor necrosis factor-α (TNF- α), leukotriene B4, IL-1β, IL-6, IL-8, as well as C-C motif chemokine ligand (CCL) 2, CCL3, and CCL4 [9].

Building from these premises, it becomes clear that exploring alarmin-related pathways is crucial for unravelling the mechanisms of inflammatory processes, particularly in a condition characterized by hyperinflammation such as CSU. Consequently, recognizing alarmins as central players in CSU could advance research aimed at developing targeted treatments directly addressing the primary causes of cutaneous inflammation.

Through a synthesis of recent findings from preclinical and clinical studies, we aim to provide a deeper understanding of the complex interaction between alarmins and CSU, focusing on immunological cellular mechanisms, to shed light on novel potential therapeutic approaches, with the final ambition of offering patient-tailored therapies.

## 2. Materials and Methods

To narratively review the literature focused on the pathogenic role of alarmins in CSU, we evaluated English-language papers published up to 10 November 2024, using the databases PubMed, Scopus, and Web of Science, as well as clinical studies registered in ClinicalTrials.gov and the EudraCT Public website. The following Medical Subject Heading (MeSH) terms were used: “alarmins”, “nuclear alarmins”, “granule-derived alarmins”, “cytoplasmic alarmins”, “IL-33”, “IL-25”, “TSLP”, “IL-1α”, “HMGB1”,“HMGN1”, “Heat-Shock proteins”, “HSP”, “adenosine 5′-triphosphate”, “ATP”, “S100 proteins”, “calprotectin”, “eosinophil-derived neurotoxin”, “defensins”, “granulysin”, “cathelicidin”, and “uric acid”, which were individually associated with: “urticaria”, “chronic spontaneous urticaria”, “CSU”, and “chronic urticaria”. Based on this selected search, we considered clinical trials, observational studies, systematic reviews, narrative reviews, and meta-analyses, while letters to the editor, editorials, expert opinions, and abstract conferences were excluded. All articles were read, and other relevant papers were identified by cross-referencing the selected articles. In addition, we analyzed studies that evaluated the possible effects of old and new therapies in the treatment of CSU on alarms. No ethical approval was required for this review.

## 3. Results

### 3.1. Epithelial-Derived Alarmins (IL-33, IL-25, TLSP)

#### 3.1.1. Interleukin-33 (IL-33)

IL-33 is an intracellular protein expressed by epithelial and endothelial cells and released into the extracellular space in response to noxious stimuli. IL-33 acts both as a pro-inflammatory cytokine and as an intracellular nuclear factor in transcriptional regulation. IL-33 binds to the ST2 receptor, a protein expressed on T helper type 2 (Th2) cells, and promotes Th2-type responses, with pro-inflammatory effects in several pathologies [12]. In a murine model of asthmatic inflammation, in vivo and in vitro tests showed that a soluble form of ST2 (sST2) antagonized the interaction between IL-33 and the membrane receptor (ST2L) [13].

An interesting role of IL-33 is its involvement in the so-called “IL-33/IL-31 axis” [14], a concept born shortly after the discovery of these cytokines, that underlines the close relationship between these two molecules. Their involvement in the inflammatory mechanisms underlying immuno-allergic diseases such as CSU is based on the theory that sees the activity of one interleukin as an inducer of the other, with consequent amplification of the inflammatory process and worsening of the damage [15]. Indeed, IL-33 promotes IL-31 release via IL-4/STAT6 and IL-33/NF-kB signaling. IL-31 activity is inhibited by suppressor of cytokine signaling (SOCS)3 [16,17].

The first human studies to investigate the role of IL-33 in CSU focused on looking for differences in alarmin concentrations in biological samples. 

In 2013, Puxeddu et al. [18] dosed (ELISA) serum levels of IL-33 and sST2 in 73 CSU patients and 40 healthy subjects as controls. Furthermore, all the patients underwent autologous serum skin testing (ASST). The researchers detected no differences in IL-33/sST2 levels between CSU and controls or between ASST positive or negative subgroups, and there was no correlation with disease activity (UAS7).

Similarly, in 2013, Metz et al. [19] analyzed a total of 328 CSU patients grouped according to severity (mild, moderate and severe), comparing their serum levels of IL-33 with healthy control subjects. No differences were found.

Consistent with the previous reports by Puxeddu and Metz, in 2017, Zheng at al. [20] found similar percentages of IL-33 between CSU patients and healthy subjects, which were unique from the results obtained for patients with acute urticaria, indicating that a Th2 response may not be important in chronic urticaria pathogenesis.

In contrast to the previous findings of Puxeddu, Metz, and Zheng, in 2017, Lin W et al. [8] reported higher plasma IL-33 concentrations in 51 CSU patients (*p* < 0.001) than in 20 controls. Stratification of patients according to disease severity (UAS7) revealed that the most severe patients had significantly higher IL-33 concentrations than the less-severe patients (*p* = 0.026). In addition, the researchers found higher IL-33 levels in the total IgE-positive group compared to the negative group (*p* = 0.010).

The complexity and contrast results of previous studies were summarized by Topal et al. [21], who indicated the necessity of further studies to better understand the possible pathogenic role of IL-33 in CSU.

The conflicting data on IL-33 may depend on its dual function, as hypothesized by Vadasz et al. [22]. Indeed, IL-33 can promote inflammation by stimulating mast cells, macrophages, and lymphocytes. On the other hand, it promotes both regulatory T and B cells (Treg and Breg, respectively), with a suppressive effect.

To investigate the pathogenic role of IL-33 in CSU, in 2015, Kay et al. [23] studied skin biopsies from CSU patients and documented a higher number of IL-33+ cells in the lesion dermis compared to both lesion-free skin areas (*p* = 0.002) and controls (*p* = 0.001). The following year, Moy et al. [24] observed that Th2 cells and Th17 cells were significantly more represented in skin biopsies from patients with chronic urticaria compared to normal skin.

To further understand the pathogenic role of some alarmins, such as IL-33, recently, Dobrican-Băruța et al. [7] compared both clinical and laboratory findings between CSU patients and healthy subjects. Receiver operating characteristic (ROC) analysis provided an area under the curve (AUC) of 0.9658 for IL-33, attributing to the serum alarmin levels to a strong ability to differentiate between CSU patients and healthy subjects (*p* < 0.0001). Furthermore, the researchers found a relevant positive relationship between serum IL-33 levels and disease severity (UAS7, r = 0.7510 with *p* < 0.0001)/impact on quality of life (Dermatological Life Quality Index (DLQI), r = 0.7981 with *p* < 0.0001).

These findings support the role of IL-33 as a potential diagnostic and prognostic biomarker in CSU and, consequently, as a potential therapeutic target. 

IL-33 is also involved in the regulation of chronic itch in CSU as well as in other skin diseases associated with severe pruritus (such as atopic dermatitis and parasitic diseases) [25]. The pruritogenic action can be exerted directly on primary sensory neurons (so-called itch receptors) or by modulating sensitivity to other Th2 inflammatory mediators of itch.

Impairment of the anatomical–functional integrity of the skin causes epithelial stress, with consequent keratinocyte release of alarmins such as IL-33, IL-25, and TSLP [26].

In turn, IL-33 directly stimulates transient receptor potential ankyrin 1 (TRPA1)+ sensory neurons. Such neurons express the related receptors ST2 [27,28].

Recently, Trier et al. [29] studied the role of IL-33 in histaminergic pruritus typically seen in CSU. Both mouse models and skin biopsy samples from CSU patients were evaluated and compared with data from healthy subjects. The induction of histaminergic pruritus also occurred independently of IL-33 signaling in sensory neurons. Furthermore, skin biopsies from patients showed increased IL-33R expression compared to healthy subjects. Admirably, the authors hypothesized that the poor response to antihistamines (AHs) observed in CSU may partially depend on the IL-33-induced enhancement of the pruritogenic capacity of histamine.

Indeed, although AHs are the cornerstone of CSU treatment, the low response rates, both with standard and high doses, reflect the difficulty in controlling symptoms [30] and motivate the scientific community in its global effort to obtain a definitive understanding of the underlying pathogenic mechanisms.

Proceeding in this direction, in 2023, Kulumbegov et al. [31] demonstrated that serum IL-33 levels in AH-resistant CSU patients were higher than the values observed in AH responders (*p* = 0.007), identifying the serum IL-33 value as a possible biomarker of pharmacological response.

#### 3.1.2. Interleukin-25 (IL-25)

IL-25 is an epithelial-derived alarmin; a member of the IL-17 family that promotes type 2 T helper (Th2) cell responses, being primarily involved in allergic and inflammatory conditions [32]. By binding to the receptor complex IL-17RA/IL-17RB, it activates downstream signaling pathways involving group 2 innate lymphoid cells (ILC2) and macrophages, which, once activated, increase the production and release of IL-13 [33]. The latter, in turn, stimulates keratinocyte proliferation and chemokines release, which recruit immune cells, including eosinophils. Furthermore, IL-25 stimulates neutrophil recruitment through macrophage activation with a p-38-dependent mechanism [34]. From these premises emerges the central role of IL-25 in the onset and exacerbation of a plethora of allergic inflammatory responses, including asthma, atopic dermatitis, and urticaria. Moving to CSU, a recent study measured serum levels of the three major epithelium-derived cytokines (IL-33, IL-25, and TSLP) in 50 CSU patients and 38 healthy controls, aiming to explore their roles in disease severity, measured by the Urticaria Activity Score (UAS7), and the impact on the Dermatology Life Quality Index (DLQI). The results showed a weak correlation between serum IL-25 levels, UAS7, and DLQI. Specifically, although IL-25 levels were found to be elevated in CSU patients compared to the control group, this increase did not reach statistical significance (*p* = 0.0823), thus suggesting a limited role of this alarmin in directly influencing CSU severity and patients’ quality of life. Furthermore, the diagnostic potential of IL-25 to distinguish CSU patients from healthy controls was assessed based on a Receiver Operating Characteristic (ROC) curve analysis, which yielded an area under the curve (AUC) of 0.6511, finally highlighting the poor diagnostic ability of IL-25 [7].

A step forward in investigating the role of IL-25 was made by Kay et al. [23], who examined the presence and localization of Th2 cytokines (IL-25, IL-33, and TSLP) in lesional versus non-lesional skin samples of eight CSU patients based on skin biopsies compared to healthy controls. Through immunohistochemistry and confocal microscopy, IL-25 levels were quantified in cutaneous tissues, along with their co-localization with various immune cells, including mast cells and eosinophils, aiming to identify possible sources of IL-25 at lesional sites. Despite a small sample size, IL-25 expression was found to be five-fold higher in lesional skin compared to non-lesional and control samples (*p* = 0.01 for non-lesional skin and *p* = 0.0009 for controls). Regarding localization, IL-25 was mainly localized in epithelial cells, mast cells, and eosinophils, which confirmed the role of this cytokine in the Th2-related inflammatory cascade. Interestingly, a positive correlation was found between IL-25 and IL-33 expressions, suggesting that these alarmins may act synergistically in the pathogenesis of CSU [23]. In conclusion, although IL-25 levels were elevated in CSU patients, both in serum and biopsy specimens [7,23], its role as an independent biomarker for CSU remains inconclusive due to its limited correlation with clinical severity (UAS7) or DLQI metrics. The results available to date further highlight the intricate inter-alarmin interactions in the onset and severity of CSU, suggesting that IL-25 may contribute to pathogenesis through indirect or synergistic pathways with other epithelial-derived cytokines, especially IL-33, rather than as a primary mediator. Finally, its potential as a therapeutic target may be hypothesized by its interaction with other Th2 cytokines and association with inflammatory cells in lesional skin, especially in patients resistant to first-line therapeutical options (oral antihistamines) or biologicals (omalizumab) currently available on the market.

#### 3.1.3. Thymic Stromal Lymphopoietin (TSLP)

TSLP, a cytokine belonging to the IL-17 family, exerts its primary functions in various body districts including the skin. Its recruitment and activation of dendritic cells (DCs) promotes the development of Th2 cells. Mast cell degranulation promoted by TSLP occurs through stimulation in the presence of IL-1 and TNF with the release of proinflammatory cytokines/chemokines [35]. In 2009, Harada et al. identified long-form and short-form isoforms for TSLP (lfTSLP and sfTSLP, respectively) in human bronchial epithelial cells [36]. The two isoforms perform opposite functions. lfTSLP promotes inflammation through binding to the functional, high-affinity receptor of the lfTSLP isoform comprising both the TSLP receptor (TSLPR) and the alpha chain of the IL-7 receptor [37,38], present on various cells such as DCs, mast cells, and eosinophils [39,40,41]. Instead, sfTSLP has homeostatic, anti-inflammatory, and antimicrobial functions.

Growing evidence supports the pathogenic role of lfTSLP in cutaneous allergic and immune-mediated diseases including CSU [35,42].

**Figure 1:**
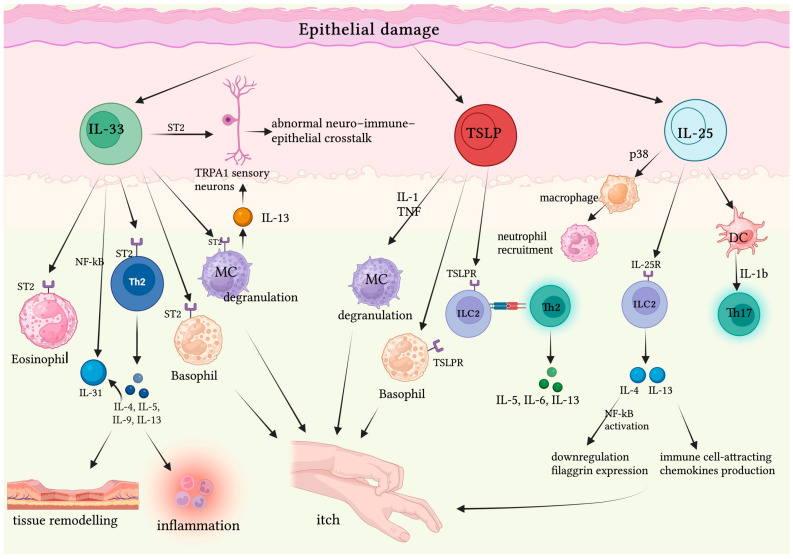
After release, IL-33 promotes type 2 immune cell activation, including Th2 cells, MC, basophils, and the consequent production of Th2 cytokines, thus enhancing inflammation and tissue remodeling. IL-33 is also involved in the IL-33/IL31 axis: IL-33 promotes IL-31 release via IL-4/STAT6 and IL-33/NF-kB signaling. IL-33 directly stimulates transient receptor potential ankyrin 1 (TRPA1)+ sensory neurons, expressing the related receptors ST2, thus leading to abnormal neuro–immune–epithelial crosstalk. IL-33 amplifies histaminergic itch in sensory neurons: stimulated by IL-33, MCs significantly increase IL-13 levels and, binding IL-13R on sensory neurons, exacerbate histaminergic itch through IL-13-dependent mechanisms. TSLP induces MC degranulation in the presence of IL-1 and TNF and enhances the production and release of proinflammatory cytokines/chemokines, including IL-5, IL-13, and IL-6. Moreover, TSLP is involved in CSU’s pruritic symptoms through basophil activation. IL-25′s main targets are dermal ILC2s, which, once activated, promote IL-4 and IL-13 release. The latter, in turn, induces keratinocyte proliferation and produces immune cell-attracting chemokines, while down-regulating keratinocyte filaggrin expression synergistically with IL-4, thus exacerbating skin barrier defects. IL-25 also promotes neutrophil recruitment via macrophage activation in a p38-dependent mechanism. IL-25 induces dermal DCs to release IL-1b, directly activating Th17 cells. Created with BioRender.com.

Patients with CSU typically present higher serum TSLP levels than healthy controls [7] and increased numbers of TSLP+ cells in the lesion dermis compared to non-lesional dermis and controls [23]. The correlation of serum levels of this alarmin with disease severity (UAS7) and QoL (DLQI) is less robust (Figure 1) [7].

### 3.2. Role of Non-Epithelial-Derived Alarmins (HSPs, SP100, EDN, Uric Acid, β-Defensins)

#### 3.2.1. Heat Shock Proteins (HSPs)

The family of heat shock proteins (HSPs) up-regulated following multiple stress stimuli (e.g., heat shock) is grouped based on size, expressed in kilodaltons (kDa), and function into high-molecular-weight (e.g., HSP70) and low-molecular-weight (e.g., HSP10) HSPs [43,44].

HSPs can prevent the aggregation of misfolded proteins and promote their degradation via proteases or autophagy, exerting a cytoprotective function in stress situations synergistically with immune responses [45,46].

Recently, studies on CSU pathogenesis have also explored the potential role of HSPs [47].

Among the larger HSPs with molecular weights ranging from 70 to 90 kDa and dependence on adenosine triphosphatase (ATPase) activity for most of their functions, HSP70 is the most-preserved molecular chaperone and best-characterized group of HSPs.

Kasperska-Zając et al. [47] studied the circulating concentrations of HSP70 and antibodies directed against HSP70 in CSU patients versus healthy subjects. The CSU patients showed higher plasma HSP70 concentrations than the healthy subjects.

Furthermore, plasma HSP70 concentrations were higher in patients with moderate to severe cutaneous conditions than healthy subjects, but this difference was not confirmed when compared to patients with mild disease activity. Similarly, CSU patients showed higher serum concentrations of anti-HSP70 antibodies than controls, which was related to systemic inflammation evaluated based on serum C-reactive protein concentration [47].

Previously, Asea et al. revealed the possibility of HSP, as an extracellular regulatory protein of monocyte activity, to play both chaperone and cytokine roles [48]. However, it is important to keep in mind that CSU is characterized by up-regulation of inflammatory cytokines such as IL-6 [49]. Therefore, more research is needed to clarify whether the up regulation of HSP70 reflects its anti-inflammatory or pro-inflammatory properties, or whether this is simply an epiphenomenon.

HSP10, a mitochondrial co-chaperone, cooperates with HSP60 in protein folding and aggregation [50]. In humans, it is also detectable in circulation, with increased levels under stress conditions [51,52]. 

Notably, HSP10 is considered a protective agent for its anti-inflammatory action that occurs through multiple pathways: (1) reduction of the expression of adhesion molecules and integrins [53], (2) inhibition of infiltration of effector T cells, typically present in the inflammatory wheal infiltrate that express the platelet activating factor (PAF) receptor [54], (3) inhibitory action on inflammatory cytokines such as IL-6 and TNF-α [55], and (4) inhibition of processes triggered by the toll-like receptor (TLR) [56]. 

Recently, a Korean study [57] explored the potential pathogenic role of anti-HSP10 IgG in CSU. The authors identified a new autoantibody, anti-HSP10 IgG, in about 40% of patients with CSU, with a significant correlation with urticaria severity, independently of H1-antihistamine response.

Additionally, the serum of CSU patients showed reduced levels of HSP-10. The authors attributed this to both the interaction of anti-HSP10 IgG with HSP10 and the reduction of HSP10 transcription by a novel micro-RNA, miR-101-5p, over-expressed via IL-4 [58]. 

Consequently, attenuation of the anti-inflammatory effect of HSP10 and increased PAF synthesis in endothelial cells enhance the recruitment of inflammatory cells, PAF-induced mast cell degranulation, and promote histamine release via cellular over-expression of the histamine receptor (H1R) by IL-4 [59]. In addition, increased serum IL-4 levels are present in almost 70% of these patients and downregulate PAF acetyl hydrolase (PAF-AH), which is able to rapidly inactivate PAF with a reduction in the PAF/PAF-AH ratio, as previously demonstrated [60].

The final result of these complex interactions fuels the formation and persistence of the wheal, a characteristic sign of CSU type IIb characterized by IgG autoantibodies against autoantigens.

Interestingly, research literature highlights that “stress proteins” have a crucial role in autoimmunity as superantigens capable of becoming targets of the immune response [61]. Among HSPs with the role of immunodominant antigens of mycobacteria and other non-viral pathogens, there are HSPs with a molecular weight of 65 kD. In fact, these microbial HSPs share a high amino acid sequence homology between prokaryotic bacteria and human eukaryotic host cells [62]. In 1994, Izaki et found significantly increased levels of anti-mycobacterial IgG antibodies of HSP 65 (0.185 ± 0.077, *p* = 0.0028) in patients affected by CSU, with similar trend also found for HSP65 IgM levels. Based on these data, antibiotic therapy in some of these patients was effective, even though urticaria is a multifactorial disease with triggers other than bacterial infections [63].

A recent in silico model [64] demonstrated that molecular mimicry between Helicobacter pylori proteome and human HSP 60 antigen did not explain the complex relationship between this bacterium and CSU [65].

#### 3.2.2. S100 Proteins

The S100 protein family includes over 25 proteins, characterized by calcium binding, low molecular weight, and solubility in 100% ammonium sulfate at a neutral pH; the latter being responsible for the nomenclature “S100 proteins”, with differences in structure and function [66,67]. Of interest for the purposes of this review, there are S100/calgranulins specifically named S100A8, also defined as calgranulin A or myeloid-related protein (MRP) 8; S100 A9, also called calgranulin B or MRP14; the heterodimer S100A8/A9 (MRP 8/14 or calprotectin); and S100A12, also defined as calgranulin C, or Receptor for Advanced Glycation End products (RAGE)-binding protein (EN-RAGE). They behave as structurally and functionally homologous damage-associated molecular patterns (DAMPs).

Interestingly, they are not normally found in healthy skin but are released by neutrophils, monocytes, and DCs via TLR4 or RAGE signaling following stressful stimuli and/or tissue injury, and they perform chemoattractant and immuno-active functions [68].

The potential pathogenic role of calgranulins in cutaneous multiple disorders has been the aim of several recent studies [69,70,71,72].

However, the literature on this subset of S100 proteins in CSU is limited to a study by Zhou et al. in 2019 [73]. These researchers compared serum levels of S100A8, S100A9, and S100A12 from 51 CSU patients with those from 20 healthy subjects, finding significantly higher values in the first group and inter-relation between S100A12 levels with those of S100A8 and S100A9 (*p* < 0.05 and *p* < 0.001, respectively). Furthermore, the tested S100 proteins were inversely related to the percentage of blood basophils, with the latter likely consumed by the release of histamine. However, none of the three calgranulins correlated with severity score UAS7.

Multiple mechanisms underlying the potential involvement of these alarmins in the pathogenesis of CSU have been hypothesized by the authors.

S100 proteins bind to RAGE and TLR4, cellular receptors that activate signaling pathways such as p38 mitogen-activated protein kinase (MAPK) and nuclear factor kappa B (NF-κB), triggering the release of pro-inflammatory molecules such as IL-6, TNF-α, and IL-1β [74].

Importantly, other evidence supports elevated serum levels of these cytokines in CSU, confirming the systemic pro-inflammatory profile [75,76].

Another interesting function of S100A12 is the strong mast cell activation through histamine and molecules such as IL-6 and monocyte chemotactic protein-1, independently of RAGE expression [77], and the chemotactic activity on mast cells through G protein-coupled receptors [78]. Therefore, S100A12 could be considered a potential biomarker of mast cell activation in CSU.

#### 3.2.3. Eosinophil-Derived Neurotoxin (EDN)

Eosinophil-derived neurotoxin (EDN/RNase2), a member of the ribonuclease A superfamily, is one of the four major secretory proteins released by human eosinophilic leukocytes, although it is also localized in the granule matrix of other cells including neutrophils, monocytes, basophils, and dendritic cells (DC). This single-chain polypeptide exerts both neurotoxic functions with antiviral activity and chemotactic functions on DC via p42/44 MAPK, enhancing TH2 immune responses [79].

Compelling evidence supports the belief that EDN may be a surrogate biomarker for eosinophil activation; for example, in severe asthma [80,81], eosinophilic esophagitis [82], and cow’s milk allergy [83].

In 2020, Saleh et al. studied 50 patients with CSU, finding mean serum EDN levels that were significantly higher than those observed in healthy controls (6.92 ± 8.01 ng/mL; 3.11 ± 1.29 ng/mL, respectively, *p* = 0.012), and positively related to disease severity [84]. More recently, a similar trend was found by Gomulka and Mędrala [85].

#### 3.2.4. Uric Acid

Uric acid (UA) is the end product of purine metabolism in humans and higher primates that, at physiological doses, can act as an antioxidant by inhibiting the release of nitric oxide and free radicals produced during inflammation. However, at chronically high body concentrations, it can trigger a pro-inflammatory state and oxidative changes in adipocytes [86]. Therefore, it represents a marker of systemic inflammatory diseases such as cardiovascular [87] and chronic kidney disease [88]. High-density lipoproteins (HDL), also called “good” cholesterol, have antithrombotic, anti-inflammatory, and antioxidant properties. Recently, the serum UA to HDL ratio (UHR) has been considered as a new marker of inflammation, with increased values in inflammatory diseases such as Hashimoto’s thyroiditis [89] and steatohepatitis [90]. A recent cross-sectional study analyzed multiple biomarkers of inflammation (including uric acid, fibrinogen, D-dimer, C-reactive protein, white blood cells, and albumin) in 90 patients with CSU compared with healthy volunteers. The patients showed lower values of uric acid (4.40 ± 0.80 mg/dL versus 4.96 ± 1.36 mg/dL, *p* = 0.005) and higher values of UHR (0.0974 ± 0.0303 versus 0.0891 ± 0.0334, *p* = 0.041) [91].

#### 3.2.5. β-Defensins

Human defensins are small host defense peptides that are classified into α- and β-defensins, distinguished by the pattern of gene expression and spatial organization of three intramolecular disulfide bonds. In contrast to α-defensins, human β-defensins (HBD) are produced primarily by epithelial cells, keratinocytes, and macrophages in response to pathogens interaction, pro-inflammatory cytokine release, or wound healing, historically serving as natural antibiotics. HBD2 indirectly impacts allergic reactions by enhancing mast cell activation and degranulation. Elevated serum levels of HBD2 have been observed in dermatological and allergic conditions, including atopic dermatitis and psoriasis [92]. Recently, Tra Cao et al. [93] investigated the relationship between serum HBD2 concentrations and clinical manifestations in 124 CSU patients compared with healthy subjects. The patients showed higher median serum HBD2 concentrations, especially in the case of CSU associated with angioedema, allowing the authors to hypothesize a possible pathogenetic role (Table 1) [93].

## 4. Discussion

Beyond the well-known role of MCs, multiple epithelial and non-epithelial-derived cytokines appear to be involved in CSU pathogenesis through an interplay that contributes to the onset of a complex inflammatory environment.

Key epithelial-derived cytokines, including IL-33, IL-25, and TSLP, act through distinct receptors that activate independent downstream pathways, resulting in an inflammatory cascade that contributes to disease resistance to standard therapies. These cytokines have been detected at elevated concentrations in CSU patients, particularly in active lesional skin, suggesting their roles, albeit variable, in disease onset and progression.

IL-33 emerges as a dual-function cytokine that acts as both a pro-inflammatory alarmin and a modulator of immune responses. This duality in the functions of IL-33 may explain, at least in part, the variable and sometimes contradictory results observed in studies dealing with its potential role in CSU onset [7,8,18,19,20,23,29,31]. Recent studies have shown elevated IL-33 levels in CSU patients compared to healthy controls, suggesting a possible role as a disease biomarker and a potential correlation between symptom severity. IL-33, therefore, may represent not only a pivotal molecule in CSU pathophysiology but also a possible therapeutic target in managing refractory cases [31].

IL-25 also contributes to CSU onset, although its role as an independent biomarker appears to be less direct. While elevated IL-25 levels have been documented in serum samples and lesional skin [7,23], their weak correlation with disease severity scores and patient-reported quality of life measures suggests a nuanced role for IL-25. 

TSLP completes this inflammatory landscape. In the context of CSU, TSLP may contribute to amplifying the type 2 inflammatory response involved in disease pathogenesis, as suggested by increased serum levels and TSLP+ cell counts detected in lesional skin [7,23]. However, its correlation with clinical severity (UAS7) and quality of life indicators (DLQI) is inconsistent, highlighting that its role in CSU is still poorly defined, and its specific cellular interactions are still subjects of research.

From this evidence, it emerges that epithelial-derived cytokine inflammatory pathways orchestrate a Th2-oriented environment that initiates and perpetuates the inflammatory response in CSU, worsening the itchy symptoms and thereby reducing the efficacy of standard oral antihistamine treatments. Since these inflammatory pathways appear to act independently, therapeutic interventions that selectively target each alarmin are of growing interest, with initial research underway to explore new therapeutic strategies.

Although no clinical trials of IL-33 inhibitors in CSU are ongoing, phase 2 studies are currently investigating the use of anti-IL-33 antibodies in other Th2-mediated inflammatory diseases such as asthma and chronic obstructive pulmonary disease (COPD), which share some immunopathological features with CSU. Specifically, the monoclonal antibodies itepekimab and astegolimab (both targeting IL-33 and its receptor ST2) have shown promising results in reducing asthma exacerbations and airway inflammation [94,95]. These preliminary positive outcomes may pave the way for the use of the same molecules for CSU treatment, thus interrupting the cycle of chronic inflammation that complicates the management of this Th2-dominated disease [96].

On the contrary, to date, research aimed at selectively blocking IL-25 has not yet developed, perhaps due to its marginal role in the pathogenesis of CSU.

Further research is required to examine TSLP inhibition. Tezepelumab, a monoclonal antibody preventing TSLP-TSLPR interactions, represents a novel approach aimed at stopping the alarmin’s pathway. Although still under investigation, the use of tezepelumab in clinical practice in CSU is not far off, also given its success as a therapy for other Th2-related conditions, representing a possible novel targeted approach for patients with CSU that is resistant to standard therapies [97].

Specifically, a phase 2b INCEPTION study (NCT04833855) evaluated tezepelumab’s efficacy in biologic-naïve patients with CSU inadequately controlled on antihistamines, comparing it with a placebo and omalizumab. Improvements in UAS7 after tezepelumab administration were detected, although the differences were not statistically significant compared to the placebo at the 16-week follow-up. However, in exploratory analyses at 32 weeks, tezepelumab demonstrated a sustained effect, showing a greater reduction in UAS7 compared to the placebo, thus suggesting a potential lasting impact and immunomodulatory effect on disease activity, even after stopping the treatment [98]. 

Interestingly, the so-called non-epithelial alarmins, including HSPs, S100 proteins, EDNs, defensins, and uric acid, could also contribute to the implementation of new therapeutic strategies in CSU. In fact, these molecules have a known protective and cellular homeostatic role in response to stressful stimuli and are overexpressed in inflamed tissues with immunogenic effects [99].

The clinical observation of significantly higher levels of HSP70 [47], anti-HSP70 antibodies [47], anti-mycobacterial HSP65 antibodies (IgG and IgM) [63], S100 proteins [73], EDN [84,85], and HBD2 [93] in cross-sectional studies enrolling CSU patients compared to healthy subjects supports their involvement in CSU pathogenesis.

However, this evidence is influenced by several methodological aspects. First, we have no data from longitudinal studies assessing serial determinations of concentrations of these alarmins and their autoantibodies. Furthermore, the cellular sources of these molecules are often not specified, and determinations are not performed on large cohorts of patients. Finally, except for the assessment of itching in response to histamine after IL-33 or saline stimulation in skin biopsies from CSU subjects, healthy subjects, and biopsies from knockout mice [29], further data from preclinical animal models did not emerge from this literature review. This paucity of data could be partly explained by the lack of an appropriate animal model of CSU, making it necessary to resort to other strategies such as mathematical models [100] in addition to human studies. This gap could further affect the molecular mechanistic understanding of this chronic inflammatory disease.

Making the understanding of the pathogenic contribution of non-epithelial alarmins in CSU even more complex is the dual behavior of some chaperonins, such as HSP70, and the current impossibility of adopting the values of all autoantibodies investigated as surrogates of disease severity.

In fact, HSP70 is an example of a chaperonin with dual action in CSU. Depending on its localization (intra- or extracellular) and the possible action of self-antigen, it can stimulate pro-inflammatory cytokines via NF-κB signaling and induce the production of autoantibodies (anti-HSP70) that amplify this inflammatory response [47,48,101], also through the possible accumulation of HSP–antibody immune complexes at the inflammatory site, as observed in placental tissues [102] and cardiovascular pathologies [103]. On the other hand, HSP70 can immuno-mitigate the inflammatory response through regulatory T-cells via TLR2 signaling pathways [101,104].

A further interesting phenomenon with potentially immunomodulatory implications is molecular mimicry: a process that could underlie the findings of increased serum levels of anti-mycobacterial antibodies HSP65 in patients with CSU in whom urticaria is related to bacterial infection [63].

The role of HSP10 in the multifaceted immunological network of the autoimmune form of CSU (type IIb) is better understood. HSP10 is considered a protective agent for its anti-inflammatory action and modulation of PAF metabolism; thus, recombinant HSP10 proteins (such as CBio Ltd., also called XToll, under license from the University of Queensland, Australia) or therapies that increase HPS10 production/secretion—therapeutic strategies already proposed for other chronic inflammatory diseases [55,105,106]—could be potential alternative treatments for patients who do not respond to antihistamines [57].

On the other hand, the role of S100 proteins and specifically of calgranulins A, B, and C in the pathogenesis of the inflammatory infiltrate of CSU remains uncertain [73]. However, following the example of several experiments that support a pro-inflammatory profile of these alarmins in other inflammatory cutaneous pathologies [107,108,109,110], the antibodies that block these proteins could also be part of the range of effective therapies used to treat this complex pathology.

## 5. Conclusions

The collection of articles dealing with alarmins suggests significant implications for future therapeutic strategies in CSU, particularly in the context of precision medicine. Through a greater understanding of how these molecules contribute to the complex inflammatory environment in CSU, biomarkers predictive of individual responses to targeted therapies could be identified.

Finally, by stratifying patients based on their cytokine profiles, those who are most likely to benefit from alarmin blockade or combination therapies could be detected, thus inhibiting multiple points along the Th2-mediated inflammatory pathway.

Future research should focus on developing diagnostic tools that assess cytokine signatures in CSU patients, enabling more patient-tailored therapeutic strategies that go beyond the current, unfortunately limited, antihistamine-based approach to address the unique inflammatory drivers of each patient’s disease.

## Figures and Tables

**Table 1:** Studies on alarmins involved in chronic spontaneous urticaria.

Alarmins	Author (Country)	Type of Experiments	N°Patients/NCs	Levels of Alarmins in CSU	Potential Biological Effects in CSU	Year [Ref.]
Epithelial-derived Alarmins						
IL-33	Puxeddu I. (Italy)	In vivo (humans)	73/40	No detected differences between the two groups (in serum)	No role in pathogenesis of CSU	2013 [18]
	Metz M. (Germany)	In vivo (humans)	30/10	No detected differences between the two groups (in serum)	No role in pathogenesis of CSU	2013 [19]
	Zheng R. (China)	In vivo (humans)	28/28	No differences in serum IL-33 values	Th2 response may not be important in CSU pathogenesis	2017 [20]
	Lin W. (China)	In vivo (humans)	51/20	↑ IL-33 (in plasma)	Possible pathogenic involvement	2017 [8]
	Kay AB (UK, Germany)	In vivo (humans)	8/9	↑ IL-33 (in lesional cutaneous biopsies)	Role of IL-33 in inflammatory responses	2015 [23]
	Dobrican-Băruța CT (Romania)	In vivo (humans)	50/38	↑ IL-33 (serum)	IL-33 as a possible biomarker in CSU	2024 [7]
	Trier AM (St Louis; New York; Baltimore; Denmark; China)	In vivo (mouse) and in vitro (mouse and humans)	NA	↑ IL-33 and IL-33R (in skin/epidermal biopsies from mice and humans)	IL-33 potentiates histaminergic itch	2024 [29]
	Kulumbegov B (Georgia, Israel)	In vivo (humans)	68/20	↑ IL-33 in antihistamine-resistant patients	IL-33 as marker of antihistamine resistance	2023 [31]
TSLP	Dobrican-Băruța CT (Romania)	In vivo (humans)	50/38	↑ TSLP (in serum)	Possible pathogenic involvement	2024 [7]
	Kay AB (UK, Germany)	In vivo (humans)	8/9	↑ TSLP (in lesional skin biopsies)	Possible pathogenic involvement	2015 [23]
	Metz M (Germany)	In vivo (humans)	62/10	No detected differences between CSU and healthy subjects (in serum)	Not useful as a biomarker of CSU	2013 [19]
IL-25	Dobrican-Băruța CT (Romania)	In vivo (humans)	50/38	No statistically significant differences	Marginal role of IL25 in CSU	2024 [7]
	Kay AB (UK, Germany)	In vivo (humans)	8/9	↑ IL-25 (in lesional skin biopsies)	Role of IL25 in the pathogenesis of CSU	2015 [23]
Non-Epithelial-derived Alarmins						
HSPs	Kasperska-Zając (Poland)	In vivo (humans)	58/22	↑ HSP70 ↑ anti-HSP70 antibodies ↑ CRP concentration (in serum)	Enhanced anti-Hsp70 antibody expression correlates with the grade of inflammation; Unclear role of HSP-70: anti-inflammatory, pro-inflammatory, or epiphenomenal effects?	2018 [47]
	Choi, B.Y. South (Korea)	In vivo (humans) In vitro	86/44	↑ anti-HSP10 IgG ↓ HSP10 ↑ IL-4 and PAF ↑ MiR-101-5p (in serum)	HSP10 has anti-inflammatory effects; Possible role of severity biomarker for anti-HSP10 IgG	2023 [57]
	Izaki S. (Japan)	In vivo (humans)	30/9	↑ anti-HSP65 IgG ↑ anti-HSP65 IgM (in serum)	Potential immunomodulation by mycobacterial HSP65 in CSU, occasionally infection-induced	1994 [63]
	Sánchez Caraballo A. (Colombia, USA)	In silico	NA	No identity between HSP60 and Helicobacter p. proteome	Low probability of molecular mimicry	2023 [64]
S100 proteins	Zhou QY (China)	In vivo (humans)	51/20	↑ S100A8, S100A9, and S100A12 (in plasma); No significant relationship with UAS7; Significant inverse correlation with basophils	S100 proteins as potential biomarkers in CSU	2019 [73]
EDN	Saleh AA (Egypt)	In vivo (humans)	50/30	↑ EDN (in serum); Positive correlation between serum EDN levels and the severity of the disease	Possible pathogenic involvement, with therapeutic implications	2020 [84]
	Gomułka K (Poland)	In vivo (humans)	15/15	↑ EDN (in serum)	Possible pathogenic involvement	2022 [85]
Uric Acid	Metin Z (Turkey)	In vivo (humans)	90/90	↓ uric acid ↑ UHR (in serum)	UHR as a promising research area for CSU	2024 [91]
β-Defensins	Tra Cao TB (Republic of Korea)	In vivo (humans)	124/56	↑ HBD2 levels (in serum), especially in cases of CSU + angioedema	Potential role of HBD2 in pathogenesis of CSU accompanying angioedema	2021 [93]

NCs, healthy normal controls; CSU, chronic spontaneous urticaria; NA, not applicable; IL-33R, IL-33 receptor; HSPs, heat shock proteins; CRP, C-reactive protein; PAF, platelet-activating factor; miR, microRNA; UAS7, urticaria activity score over 7 days; EDN, eosinophil-derived neurotoxin; UHR, uric acid/HDL ratio; HBD2, human beta defensins 2.

## Data Availability

Not applicable.
